# Predictors of early recurrence after resection of colorectal liver metastases

**DOI:** 10.1186/s12957-015-0549-y

**Published:** 2015-04-01

**Authors:** Ricky Harminder Bhogal, James Hodson, Simon Roderick Bramhall, John Isaac, Ravi Marudanayagam, Darius Feroze Mirza, Paolo Muiesan, Robert Peter Sutcliffe

**Affiliations:** University Hospitals of Birmingham, Metchley Lane, Birmingham, West Midlands B15 2TT UK; Centre for Liver Research, Institute for Biomedical Research, The Medical School, University of Birmingham, Edgbaston, Birmingham, West Midlands B15 2TT UK

## Abstract

**Background:**

Early recurrence after resection of colorectal liver metastases (CLM) is common. Patients at risk of early recurrence may be candidates for enhanced preoperative staging and/or earlier postoperative imaging. The aim of this study was to determine if there are any risk factors that specifically predict early liver-only and systemic recurrence.

**Methods:**

Retrospective analysis of prospective database of patients undergoing liver resection (LR) for CLM from 2004 to 2006 was undertaken. Early recurrence was defined as occurring within 18 months of LR. Patients were classified into three groups: early liver-only recurrence, early systemic recurrence and recurrence-free. Preoperative factors were compared between patients with and without early recurrence.

**Results:**

Two hundred and forty-three consecutive patients underwent LR for CLM. Twenty-seven patients (11%) developed early liver-only recurrence. Dukes C stage and male sex were significantly associated with early liver-only recurrence (*P* < 0.05). Sixty-six patients (27%) developed early systemic recurrence. Tumour size ≥3.6 cm and tumour number (>2) were significantly associated with early systemic recurrence (*P* < 0.001).

**Conclusions:**

It is possible to stratify patients according to the risk of early liver-only or systemic recurrence after resection of CLM. High-risk patients may be candidates for preoperative MRI and/or computed tomography-positron emission tomography (CT-PET) scan and should receive intensive postoperative surveillance.

## Background

The liver and lungs are the most frequent sites of distant metastases from colorectal cancer (CRC). Following diagnosis, 50% to 60% of patients with CRC will develop colorectal liver metastasis (CLM) [1], and 11% will develop pulmonary metastasis [2]. Surgical resection is the most effective treatment for CRC that has metastasized to the liver [3] or lung [4]. Indeed, in selected patients, liver resection (LR) for CLM has yielded a median 5-year survival rate of 25% to 58% [3,5,6] and a median 5-year survival of 45% to 60% for solitary liver metastasis [3]. However, recurrence after resection of colorectal liver metastases is common, developing in the liver remnant in up to 30% of patients [7,8] and at extra-hepatic locations in up to 50% [9-12].

Despite surgical resection, the relatively high recurrence rate is likely due to occult micro-metastases. Local and/or systemic recurrence may develop within months to years after LR [13]. Early recurrence may be due to aggressive tumour biology, inadequate surgical resection and/or failure of systemic therapy and may also be an indication of suboptimal pre-operative staging. There are currently no universally agreed protocols for either preoperative imaging before LR or for surveillance postoperatively [14]. Contrast-enhanced computed tomography (CT) is the imaging modality of choice for staging patients with liver metastases and for postoperative monitoring. Magnetic resonance imaging (MRI) has a higher sensitivity than CT in detecting liver metastases, particularly when used with liver-specific contrast [15], but is not routinely performed in many centres [16]. Similarly, the role of fluorodeoxyglucose (FDG) positron emission tomography (PET) or CT-PET to identify extra-hepatic disease before LR remains unproven [17].

The risk factors for recurrence after LR are well documented and relate to the biology and stage of the primary tumour, the burden of liver metastases and the response to chemotherapy [3,5,18]. Recent studies have suggested factors that may predict early recurrence after liver resection for CLM. Vigano *et al*. have shown that T3-T4, synchronous CLM and limited resection margins increase the risk of recurrence [19]. In addition, the same group showed that adjuvant chemotherapy reduced recurrence rates. Other authors have suggested that the number of liver metastases predicts early recurrence [13]. These particular subgroup of patients may benefit from enhanced pre-operative staging and/or intensive post-operative surveillance in the early post-operative period.

Patients at risk of early recurrence may also benefit from neoadjuvant or adjuvant chemotherapy. The aim of this study was to identify predictors of early liver-only or systemic recurrence after resection of CLM.

## Methods

### Patients and data collection and statistical analysis

We reviewed our prospectively held departmental database to identify all patients who had undergone LR for CLM between January 2004 and December 2006 inclusive. Two hundred forty-three patients were identified. Patients were considered for LR after clinical evaluation and pre-operative staging with a chest, abdominal and pelvic CT scan. All patients were discussed within a specialist hepatobiliary multidisciplinary team meeting. MRI was performed selectively in patients with advanced primary tumours (T4 or N2) or synchronous metastases. CT-PET was performed in selected patients to assess any suspicious extra-hepatic lesions detected by CT.

**Figure 1:**
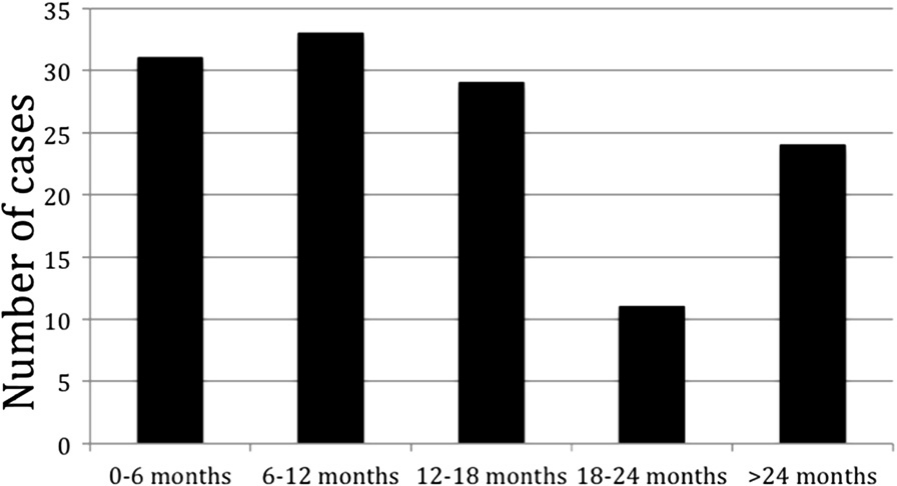
**The timing of liver-only and systemic recurrence following LR for CLM.**

After initial LR, patients underwent regular clinical assessment, serial serum CEA measurement and surveillance CT scans at 1 and 2 years postoperatively. In this study, early recurrence was defined by the presence of either liver-only or systemic (with or without liver involvement) disease within 18 months after liver resection. Eighteen months was selected as a cut-off based on an analysis of the timing and pattern of postoperative recurrence in the entire cohort. The reason for opting for this time period is illustrated in Figure 1. Most liver-only and systemic recurrence occurred with 18 months following LR.

It is unlikely that enhanced preoperative staging (that is, MRI and/or CT-PET) would detect additional sites of disease in patients who subsequently develop recurrence beyond 18 months after surgery. Potential risk factors for early recurrence were identified including clinical, radiological and pathological parameters: age, gender, site and nodal status of the primary CRC, the size and number of hepatic tumours. Pre-operative CEA was not included in the analysis due to incomplete data. Initially, for the purposes of analysis, a range of variables was compared between the three study groups (No recurrence/Liver recurrence/Systemic recurrence). For the continuous variables, a Kruskal-Wallis test was performed to test for variations between the three groups. Where this was significant, *post hoc* pairwise tests were performed between the non-recurrence group and both recurrence groups, in order to test for significant differences. A similar approach was applied to the binary variables in which a Fisher’s exact test was performed on all three groups initially, with *post hoc* tests used to compare the non-recurrent patients with patients in the other two groups. Patient survival was calculated using Kaplan-Meier estimates. *P* values <0.05 were considered statistically significant.

## Results

In this study, 243 patients underwent LR of CLM with curative intent between January 2004 and December 2006 inclusive. Table 1 shows the overall demographics of the study population.

**Table 1:** **The overall patient demographics in the study population**

**Parameters**	**Values**
Age at time of LR	
≤ 65	46%
> 65	54%
Gender	
Male	74%
Female	26%
Site of primary CRC	
Colon	57%
Rectum	43%
Dukes stage of primary CRC	
Dukes A	3%
Dukes B	26%
Dukes C	70%
Adjuvant chemotherapy	
Yes	79%
No	21%
Number of metastasis	
1	45%
2	24%
3	13%
≥ 3	18%
Distribution of metastasis	
Unilobar	74%
Bilobar	26%
Extra-hepatic disease	
Yes	96%
No	4%
Post-op resection status	
R0	81%
R1	13%
R2	6%
Type of LR	
Minor	26%
Major	74%

**Figure 2:**
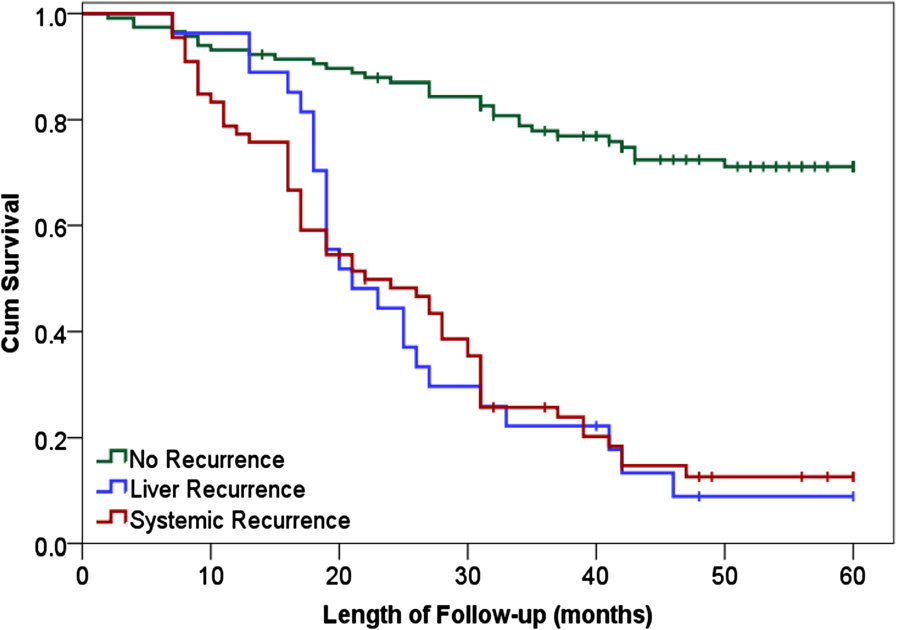
**Overall patient survival in following LR for CLM.**

At a median follow-up of 58 months (range 33 to 74 months), 93 patients (38%) developed early recurrence (defined as within 18 months of surgery), including 27 patients (11%) with liver-only recurrence and 66 patients (27%) with systemic recurrence (with or without liver recurrence). Thirty-five patients (14%) developed late recurrence and 115 patients (47%) were recurrence-free at follow-up (Table 2). Median times to diagnosis of recurrence in patients with liver-only recurrence and systemic recurrence were similar: 11 [6-14] *vs*. 9.5 [6-14] months (*P* = 0.841). In patients with early liver-only recurrence, 19 patients (70%) had treatable lesions (repeat LR 11, radiofrequency ablation 8), and 8 patients were suitable for palliative treatment only. Seventeen patients (26%) with early systemic recurrence were amenable to further surgery (pulmonary metastasectomy, *N* = 13) or ablation (*N* = 4). Twenty-seven patients (41%) received palliative chemotherapy and the remaining 22 (33%) were suitable for best supportive care only. Five-year overall and disease-free survival rates in the entire cohort were 47% and 42%, respectively. Median survival in patients with disease recurrence (liver or systemic) was 6.5 months (range 2 to 26 months). As expected, disease recurrence was associated with significantly worse overall survival (Figure 2).

**Table 2:** **Demographics of patients with no recurrence and those with liver-only and systemic recurrence**

		**No recurrence**	**Early liver-only recurrence**	**Early systemic recurrence**
		**(** ***n*** **= 115)**	**(** ***n*** **= 27)**	**(** ***n*** **= 66)**
Male–female ratio		1:0.4	1:0.13	1:0.4
Age (years)		65	67	66
(Quartiles)		(56 to 71)	(65 to 73)	(58 to 71)
Primary tumour site CRC (%)	Colon	63	71	70
	Rectum	37	29	30
Primary tumour stage (%)	Dukes A	11	0	4
	Dukes B	25	4	22
	Dukes C	64	96	74
Chemotherapy after colectomy (%)		92	90	94
Pre-operative staging modality (%)	CT	94	96	91
	MRI	5	4	7
	Other	1	0	2
Post-operative staging modality	CT	88	91	87
	MRI	10	9	10
	Other	2	0	3

Analysis of our patient cohort revealed that male patients and advanced stage primary tumours (Dukes C) were significant risk factors for early liver-only recurrence (see Table 3). Early systemic recurrence was more likely in patients with a high burden of liver metastases (tumour diameter >3.6 cm or tumour number ≥2.3).

**Table 3:** **Analysis of factors predicting liver-only and systemic recurrence in patients following liver resection for CLM**

	**No recurrence**	**Liver-only recurrence**	**Systemic recurrence**
	**(** ***n*** **= 115)**	**(** ***n*** **= 27)**	**(** ***n*** **= 66)**
Number of metastases	1	2	2.5*
Median (quartiles)	(1 to 2)	(1 to 2)	(2.3 to 3.1)
Largest tumour size	2.9	2.7	3.9*
Median (quartiles)	(2.5 to 3.2)	(2.4 to 3.2)	(3.6 to 4.2)
Male gender (%)	67	93**	56
Dukes C CRC	64	96**	74

*P < 0.05 relative to no recurrence group; **P < 0.05 relative to no recurrence group.

## Discussion

In an era of modern chemotherapy, surgical resection of CLM can be justified and may yield long-term survival in selected patients [20-22]. However, disease recurrence after LR is common and negatively impacts on patient survival [13,18,22]. Disease recurrence presumably reflects the presence of viable tumour deposits that are undetected by conventional pre-operative CT [23]. MRI is increasingly being utilized to characterize benign and malignant liver lesions [14,24-26] and appears to be more sensitive than CT, particularly when used with liver-specific contrast agents [14]. The potential advantages of MRI over CT are particularly evident in patients with background hepatic steatosis after chemotherapy [27]. However, at present, there is insufficient evidence to justify the routine use of MRI prior to LR for CLM. Risk factors for early recurrence after LR have been suggested previously such as multiple (>8) CLM [13] with recurrence within the liver being the commonest cause of treatment failure [28]. However, no studies have identified factors that specifically predispose to early liver-only or systemic recurrence. Previous multivariate analysis has revealed node positive primary tumours, advanced T stage, presence of extrahepatic disease, CEA >200 ng/ml, multiple tumours, tumour size >5 cm and short disease-free interval as predictors for early recurrence and poor overall survival [5,18,29,30]. Using this data, a clinical risk score was created to help predict which patients will benefit most from surgical intervention [5]. The current study expands these known risk factors by clearly demonstrating that larger and multiple tumours increase the risk of early systemic recurrence and male gender and advanced CRC predispose to early liver-only recurrence after LR.

The first aim of our study was to determine if there are any preoperative risk factors that may predispose patients to tumour recurrence within the liver remnant specifically within the early post-operative period. On analysis, male sex and advanced primary tumours (Dukes C) increased the risk of early liver-only recurrence. Such patients may be candidates for pre-operative MRI, and they may also benefit from enhanced postoperative surveillance. Early post-operative imaging (CT or MRI) in high-risk patients may identify liver-only recurrence at an earlier, treatable stage, which may potentially influence long-term survival although there remains no conclusive data from the available literature. Other groups also investigating the risk factors for CLM recurrence after LR have failed to show any affect of gender unlike the reported study [31]. The precise reasons underlying this male preponderance remain unclear.

The second aim of our study was to identify any potential risk factors that predict systemic recurrence specifically in the early post-operative period. Our data has indicated that patients with multiple tumours (three or more) or tumours greater than 3.6 cm are at high risk of early systemic recurrence. This group of patients is unlikely to benefit from LR as an isolated strategy and should be considered for pre-operative CT-PET and systemic chemotherapy. Using this approach, some patients with detectable FDG avid extra-hepatic disease may be spared from futile liver surgery [32]. PET/CT produces a fusion image combining conventional cross-sectional, anatomical imaging of CT with the biological, functional imaging of PET [33]. It can be utilized successfully to identify and stage primary CRC [34] as well as metastases [33] and has also been used to great effect in the staging of pancreatic [35] and lung [36] cancers. PET/CT in CLM patients may be associated with alterations in patient management in 34% owing to disease upstaging [37]. Indeed, recent meta-analyses found FDG-PET was the most sensitive method for detection of liver metastases and extra-hepatic metastatic disease with sensitivities of 90% to 92% [15,17]. FDG-PET had a significantly higher pooled sensitivity and specificity for hepatic disease and EHD when compared to CT [38]. Moreover, PET/CT identifies more definitely normal and definitely abnormal lesions than with PET alone in CRC patients with improvements in staging and restaging [34]. Indeed, these changes in staging alter patient management in 25% of patients with the use of 18FDG-PET resulting in a reduction in unnecessary surgical interventions [39]. In a recent prospective study comparing 100 CLM patients staged by conventional techniques with 103 patients staged with an additional FDG-PET, the rate of non-therapeutic laparotomies was significantly reduced in patients having preoperative FDG-PET. [38]. The results of this study were mirrored by a randomized study of 150 CLM patients selected for surgical resection by CT imaging alone or CT plus FDG-PET, which similarly demonstrated a significantly reduced rate of futile laparotomies [40]. Taken together with previous studies, it appears that liver recurrence following resection for colorectal metastasis is associated with T3-T4 disease, synchronous CLM, limited resection margins, Dukes C stage and male sex. Systemic recurrence appears to correlate with tumour size and tumour number.

## Conclusions

In summary, it is possible to identify patients at high risk of early liver-only or systemic recurrence after LR for CLM. Such patients may be candidates for enhanced pre-operative staging to detect occult metastases and may also benefit from early post-operative imaging. A tailored approach to pre-operative staging in patients with CLM warrants further evaluation in a prospective study.
